# Bioactivity of crude fucoidan extracted from *Sargassum ilicifolium* (Turner) C. Agardh

**DOI:** 10.1038/s41598-022-19370-7

**Published:** 2022-09-23

**Authors:** Min-Hsuan Tsou, Cheng-Chang Lee, Zhi-Yuan Wu, Zui-Harng Lee, Hsiu-Mei Lin

**Affiliations:** 1 Department of Bioscience and Biotechnology, National Taiwan Ocean University, Keelung, 20224 Taiwan; 2 Center of Excellence for the Oceans, National Taiwan Ocean University, Keelung City, 20224 Taiwan; 3 Center of Excellence for Ocean Engineering, National Taiwan Ocean University, Keelung City, 20224 Taiwan

**Keywords:** Analytical chemistry, Organic chemistry

## Abstract

Fucoidan derived from brown algae has been shown to exhibit antitumor and antioxidant effects, so research on sulfated polysaccharides is increasing. The purpose of this study was to evaluate the characteristics and biological activity of fucoidan that was extracted at two temperatures (65 and 80 °C) from *Sargassum ilicifolium* (Turner) C. Agardh from five regions of Taiwan. The data show that there are significant differences in the yield, sulfate and total sugar content of *Sargassum ilicifolium* (Turner) C. Agardh grown in different locations in the same sea area. HPLC was used to determine the monosaccharide compositions of the fucoidan, which contains fucose, mannose, mannose, glucose and galactose and have a low molecular weight of less than 5 kDa, and then we will select the algae collected in Fugang, Taitung, for further biological activity research. The sampled *Sargassum ilicifolium* (Turner) C. Agardh at all five locations has a good polyphenol content, and it shows great DPPH radical scavenging activity, ABTS radical scavenging activity, Ferrous ion-chelating activity and Reducing power. The *Sargassum ilicifolium* (Turner) C. Agardh that was collected from Taitung Fugang is not toxic to L929 normal cells, but for A549 cancer cells and HCT116 cancer cells, it is known from the results that it has good cytotoxicity for A549 cancer cells. Thus, this study found that the *Sargassum ilicifolium* (Turner) C. Agardh that was collected from Taitung Fugang has significant antioxidant and anticancer properties.

## Introduction

Fucoidan is a sulfated polysaccharide that is rich in fucose and can be extracted from brown algae^1^, such as *Sargassum* sp. and *Fucus* sp. At present, brown algae can mainly extract fucoidan^2^ and alginic acid^3^. Sodium alginate (NaC_6_H_7_O_6_) is a linear polysaccharide, a derivative of alginic acid, consisting of β-D-mannuronic acid M and (α-L-guluronic acid G) by pressing the (1 → 4) key to connect. Sodium alginate is a component of the cell walls of marine brown algae and contains approximately 30–60% alginic acid. Since sodium alginate is a naturally occurring anionic polymer with hydrophilicity, biocompatibility and reactivity with divalent and trivalent cations, gelation and film-forming ability, it is widely used in food, drug delivery, and pharmaceutical industry. There are potential applications in various aspects of delivery, tissue engineering, wound dressings, synthetic biofilms, and wastewater treatment. There have been many recent studies on fucoidan, mainly to evaluate its potential biological activity, including its potential for use as antitumor drugs^4^, antioxidants^5^, anti-inflammatory drugs^6^, anticoagulants^7^, antiviral drugs^8^, and immunity-regulating activity^9^. These research results show that, in addition to its original biological activity, fucoidan also has biomedical applications^10^. Taiwan is an island state that is surrounded by clear and clean seawater, so there are abundant brown algae in the intertidal zone that can be collected for extraction in the springtime. Professor Kylin of Uppsala University in Sweden confirmed the existence of fucoidan, which is a heteropolysaccharide with L-fucose and sulfate groups^11^. The chemical structure of fucoidan also includes uronic acid, galactose, xylose, mannose, rhamnose, glucose and fucose^12^. Among these listed sugars, the fucose content was highest. Fucoidan is mainly present in different brown seaweeds and marine invertebrates, such as seaweed, *Undaria pinnatifida*, *Saccharina japonica*, *Sargassum*, and Holothuroidea^13^. According to previous research, biological activity can be affected by different factors, such as structure, sulfate content, and molecular weight^14–17^. As it is difficult to identify the chemical structure of sulfated polysaccharides in algae, the relationship between their structure and biological activity is not completely clear. The outstanding biological effects of sulfated polysaccharides may be due to the presence of different amounts of sulfate groups. In addition, the position of the sulfated group along the main chain of the macromolecule plays an important role in the combination of its functional properties. Oliveira et al.^18^ showed that three fucoidan extracts (e.g., FE 1, FE 2, and FE 3) were extracted in the study, and significant differences in the sulfate positions and branches were found and indicated that FE 2 has a more branched chain, and then the experimental data indicate that the sulfate locations and branched chain numbers were determined to be important reasons for the bioactive ability of fucoidan^18^. Fucoidan, with a molecular weight in the range of approximately 10–300 kDa, the data show the strongest anticoagulant activity^19^. Alekseyenko et al.^20^ studied that Fucoidan anti-expansion algae from the Sea of Okhotsk had anti-tumor and anti-metastasis biological activity in mice with lung cancer. In 2009, Yoon showed that fucoidan is usually divided into low (< 10 kDa), medium (10–10,000 kDa) and high (> 10,000 kDa) molecular weights^21^. In 2010, Wang et al.^3^ used the principle of algae exchange column to separate brown algae (*Sacharina japonica*, formerly *Laminaria japonica*) into low molecular weight fucoidan (DFPS). These three types of fucoidan (DF1, DF2, DF3) mainly contain a variety of fucose, galactose and sulfate groups. The results show that all three fucoidans have quite good antioxidant activity, of which DF1 is the best^5^. ln this study, The structure and monosaccharide composition of algae in different locations are affected by the algae^22^, collection season^23^, age and location of the algae^23^, which affect their physiological activity. In addition, Taiwan is an island country surrounded by clear and clean waters. To the north is the "East China Sea" on the east side of the Asian continent, to the east is the world's largest ocean, the "Pacific Ocean", to the south is the "Bashi Strait" and the Philippines, and to the west is the "Taiwan" which is more than 100 km away. The Taiwan Strait is separated from mainland China by water, and the coastline is about 1200 km long. Therefore, the intertidal zone of Taiwan waters is rich in brown algae that can be collected for extraction in the springtime. Therefore, this study wanted to investigate *Sargassum ilicifolium* (Turner) C. Agardh from five locations of southern Taiwan, namely, Pingtung-Kenting, Pingtung-Tanziwan, Pingtung-Nanwan, Chuanfan Rock, Pingtung-Haikou and Taitung-Fugang was collected. Algae were extracted at two different temperatures, 65 and 80 °C, and their characteristics, antioxidant activity and cell viability assay were then analyzed.

## Materials and methods

### Materials

*Sargassum ilicifolium* (Turner) C. Agardh, ethanol (C_2_H_5_OH), calcium chloride (CaCl_2_), barium chloride (BaCl_2_), gelatin, hydrochloric acid (HCl), sodium sulfate (Na_2_SO_4_), trifluoroacetic acid (TFA), 1-phenyl-3-methyl-5-Pyrazolone (PMP), methanol (MeOH), chloroform (CHCl_3_), acetonitrile (ACN), sodium nitrate (NaNO_3_), mannose, fucose, glucose, galactose, xylose, 2,2-diphenyl-1-picrylhydrazyl (DPPH), 2,2'-azino-bis(3-ethylbenzthiazoline-6-sulphonic acid)(ABTS), potassium persulfate (K_2_S_2_O_8_), acetone (CH_3_COCH_3_), potassium chloride, KCl, Sodium hydrogen phosphate, Na_2_HPO_4_, potassium chloride (KCl), sodium hydrogen phosphate (Na_2_HPO_4_), sodium chloride (NaCl), potassium dihydrogen phosphate (KH_2_PO_4_) and thiazolyl blue tetrazolium bromide (MTT) were purchased from Sigma-Aldrich; sodium hydroxide (NaOH), toluene, and dimethyl sulfoxide (DMSO) were purchased from J.T. Baker; minimum essential media (MEM), Ham's F-12 K (Kaighn's) medium, fetal bovine serum (FBS), McCoy's 5A medium, and antibiotic–antimycotic (AA) were purchased from Gibco.

### Algae materials

In this study, we collected, extracted and analyzed *S. ilicifolium* from Tanziwan, Pin-tong (A01): 21.95157172519444, 120.77479067816147, Nanwan, Pin-tong (A02): 22.087739596082514, 120.89149059728416, Chuanfan Rock, Pin-tong (A03): 21.931320398029037, 120.82425785206844, Haikou,Pin-tong (A04): 22.092263913407553, 120.71465994995685, Fugang, Taitung (A05): 22.79114556521653, 121.19297473938583, which was collected in March 2018. The algae were provided by the Lin Showe-Mei Lab of the National Ocean University of Taiwan.

As soon as *S. ilicifolium* was collected, it was immediately brought back to the laboratory. Then, the samples were washed with clean water and dried in a large oven at 40 °C. Afterward, the samples were ground into powder with a blender and were finally passed through a 35 mesh. After sieving, we stored the samples in a − 80 °C freezer until later use.

#### Extract of fucoidan

The methods for fucoidan extraction from brown macroalgae were previously described by Chen et al.^24^. Briefly, 8 g of dry algae powder were mixed with 100 mL of 95% ethanol and then stirred for at least 12 h at room temperature to remove lipids and pigments. Next, removed the supernatant and dried the precipitate overnight at room temperature. Then, 5 g of pretreatment algae powder were mixed with 200 mL of deionized water and stirred for 1 h at 65 or at 80 °C. Afterward, the solution was centrifuged at 3500 rpm for 10 min and the supernatant was collected, 1% CaCl_2_ was added to the supernatant to precipitate the alginate and it was left to stand at 4 °C overnight. The solution was centrifuged at 3500 rpm for 10 min, and the supernatant was collected, and 95% EtOH was used for the precipitation of fucoidan. Finally, the resulting precipitate was freeze-dried to obtain the crude fucoidan.

$${\text{Extraction yield}}: \, \left( {{\text{Fa}}/{\text{Fb}}} \right) \, \times { 1}00 \, \%$$

Fa is the dry mass weight of the extracted solid

Fb is the dry weight of the algae sample

#### Chemical analysis of fucoidan

##### Determination of the total sugar content of fucoidan

The method of this section is the phenol–sulfuric acid colorimetry^25^. The measurement principle is to estimate the total sugar content based on the color reaction of the structural formula molecules with the upper environmental solution, and the calibration curve was established by the standard sample. First, 10 mg of the sample was dissolved in 10 mL of deionized water, and then 0.4 mL of the sample was taken, mixed with 20 μL of 5% phenol and 1 mL of concentrated sulfuric acid, reacted at room temperature for 20 min, and the absorbance was measured at 490 nm. (Fucose is the standard sample).

##### Determination of fucoidan sulfate content

The method was the turbidimetric method^26^. The principle is that in an acidic medium, the sulfate of the solution and the added barium ions form fine barium sulfate crystals, making the aqueous solution turbid. The turbidity is directly proportional to the sulfate content in the sample to calculate the content. First, add 0.5 g of gelatin (from bovine skin) into 100 mL of deionized water, heat and stir to dissolve at 60 °C, add 0.5 g of barium chloride solution after cooling, and store at 4 °C. Take 0.1 g of the sample and 10 mL of HCl (12 M) into the round bottom flask, heat to reflux at 100 °C for 1 h, cool to room temperature, add 0.2 mL of the solution to the microcentrifuge tube, and add 1 mL BaCl_2_-Gelatin solution, mix well for 15 min, and measure the absorbance at 360 nm. (Based on sodium sulfate as the standard sample).

#### Fourier transform infrared spectroscopy analysis (FT-IR)

Crude fucoidan was mixed with potassium bromide (KBr, 1:10) and pressed into a pellet under vacuum using a pelletizer. IR spectra were obtained using an FTIR spectrophotometer (BRUKER TENSOR Series FT-IR Spectrometer). The infrared spectrum was recorded from 4000 to 400 cm^−1^ and was compared with the background spectrum of KBr alone.

#### Monosaccharide analysis

##### Hydrolysis of fucoidan

This method was used by Dai et al.^27^. A 20 mg sample was mixed with 5 mL of 2 M TFA and then hydrolyzed at 120 °C for 4 h. After cooling to room temperature, the pH of the sample was adjusted to pH 7, water was added to form a 20 mL volume, and the sample was preserved for later use.

##### Derivatization with PMP

This method was used by Honda et al.^28^ and Zhang's et al.^1^. In a water bath at 70 °C, 1 ml of the hydrolysate was mixed with 0.5 mL of 0.03 M NaOH and 0.05 M PMP-methanol solution for 60 min. After cooling, the sample was neutralized with 0.5 mL of 0.03 M HCl, evaporated under a vacuum, and then dissolved in 1 ml of deionized water. We added 1 mL each of deionized water and chloroform to remove the excess derivatization reagent. After centrifugation (3500 rpm; 4 °C; 10 min), the supernatant was filtered through a 0.22-μm membrane, and the filtrate was collected for high-performance liquid chromatography (HPLC) analysis.

##### HPLC determination

This method is a modification of that of Dai's et al.^27^ and Wang et al.^29^. The monosaccharide compositions of crude fucoidan were determined by high-performance liquid chromatography (HPLC). HPLC analyses were performed using a Hypersil BDS-C18 column (4.6 mm × 250 mm) at a wavelength of 245 nm. The flow rate was 1.0 mL/min. A monosaccharide mixture containing fucose, xylose, glucose, galactose, and mannose was used as a standard sample.

#### Molecular weight analysis

Molecular weights (MW) were determined by high-performance liquid chromatography with a refractive index detector (HPLC-RI) and using an OHpak SB-806 M HQ column (8.0 mm × 300 mm). The flow rate was 1 mL/min. Then, a 1 mg sample was dissolved in 1 mL of deionized water and was filtered through a 0.22-μm filter. Pullulan (0.18–642 kDa) was used as the standard sample.

#### Analysis of polyphenols

To determine the polyphenol content, 2 mg of sample was dissolved in 10 mL of deionized water and analyzed using by Folin–Ciocalteu method^30^.

### Determination of antioxidant activity

#### DPPH (1,1-diphenyl-2-picrylhydrazyl) radical scavenging activity

The scavenging activity for DPPH free radicals was measured according to the method of Shimada et al.^31^. Crude fucoidan at different concentrations (0.0625^−1^ mg/mL) was prepared in deionized water, and then 1 mL of the sample was mixed with 1 mL of DPPH in methyl alcohol and incubated in the dark. After 30 min, the absorbance of the sample was measured at 517 nm using a UV–Vis spectrometer.

$${\text{DPPH scavenging activity}} = \left( {{\text{A}}_{{{\text{control}}}} - {\text{ A}}_{{{\text{sample}}}} } \right)/{\text{A}}_{{{\text{control}}}} \times 100\%$$

A_control_: absorbance of the control (1 ml of ethanol with 1 ml of the DPPH solution)

A_sample_: absorbance of the sample solution

#### ABTS [2,2'-azino-bis (3-ethylbenzthiazoline-6-sulfonic acid)] radical scavenging activity

The scavenging activity for DPPH free radicals was measured according to the method of Ree et al.^32^. Crude fucoidan at different concentrations (0.0625–1 mg/mL) was prepared in deionized water. The ABTS radical cation was produced by reacting 9.8 mL of the 7 mM ABTS solution with 0.2 ml of 2.25 mM potassium persulfate in the dark at room temperature for 16 h. Then, the ABTS radical cation solution was diluted with ethanol to reach an absorbance of 0.70 at 734 nm. To determine the scavenging activity, 25 μL of the fucoidan sample solution was first transferred into 96-well microplates. Next, 75 μL of the ABTS solution was added to the polysaccharide samples, and the reaction mixture was then incubated for 20 min in the dark at room temperature. Finally, the absorbance of the sample solution was measured at 734 nm using an ELISA (enzyme-linked immunosorbent assay) microplate reader.

$${\text{ABTS}}:{\text{ scavenging activity}} = \, \left( {{\text{A}}_{{{\text{control}}}} - {\text{ A}}_{{{\text{sample}}}} } \right)/{\text{A}}_{{{\text{control}}}} \times { 1}00\%$$

A_control_: absorbance of the control (25 μL of ethanol with 75 μL of the ABTS solution)

A_sample_: absorbance of the sample solution

#### Ferrous ion-chelating activity

The ferrous ion-chelating activity of polysaccharides was measured according to the method of Wang et al.^33^. Crude fucoidan at different concentrations (1–8 mg/ml) was prepared in deionized water. First, 200 μL of sample, 740 μL of methanol and 20 μL of FeCl_2_ solution (2 mM) were mixed homogeneously. Then, 5 mM ferrocene (40 μl) was added, and the reaction mixture was incubated at room temperature for 10 min. Finally, the absorbance of the sample solution was measured at 562 nm using an ELISA (enzyme-linked immunosorbent assay) microplate reader.

$${\text{Chelating activity}}:\left( {{\text{A}}_{{{\text{control}}}} - {\text{ A}}_{{{\text{sample}}}} } \right)/{\text{A}}_{{{\text{control}}}} \times { 1}00\%$$

A_control_: absorbance of the control (EDTA solution)

A_sample_: absorbance of the sample solution

#### Reducing power assay

Reducing power was measured according to the method of Wang et al.^33^. Crude fucoidan at different concentrations (1–8 mg/ml) was prepared in deionized water. 500 μL of sample, 500 μL of phosphate buffer (0.2 M, pH 6.6) and 500 μL of potassium ferricyanide (1%) were mixed at 50 °C for 20 min, and 500 μL of trichloroacetic acid (10%) was then added to the mixture. Finally, 500 μL of this mixture, 500 μL of distilled water and 100 μL of FeCl_3_ (0.1%) were homogeneously added, and the reaction solution was incubated at room temperature for 10 min. The absorbance of the sample solution was measured at 700 nm using an ELISA (enzyme-linked immunosorbent assay) microplate reader. Ascorbic acid was used as the standard sample.

### Anticancer activity

#### Cell culture

The methods were as previously research^34^. L929 (Mouse fibroblast) cells were purchased from ATCC, and cultured in MEM that was supplemented with 10% (v/v) fetal bovine serum (FBS) and 1% antibiotics (AA) in an incubator with 5% CO_2_ at 37 °C. A549 (adenocarcinomic human alveolar basal epithelial) cells were purchased from FIRDI, and cultured in F-12 K medium that was supplemented with 10% (v/v) fetal bovine serum (FBS) and 1% antibiotics (AA) in an incubator with 5% CO_2_ at 37 °C. HCT116 (human colorectal carcinoma) cells were purchased from ATCC, and cultured in McCoy's 5A medium that was supplemented with 10% (v/v) fetal bovine serum (FBS) and 1% antibiotics (AA) in an incubator with 5% CO_2_ at 37 °C.

#### Cell viability assay

The methods were as previously research^34^. Selection of normal cell models (e.g., L929 cells) according to ISO 10993-5, used to detect whether fucoidan damages normal cells, and two cancer cell models (e.g., A549 and HCT116 cells) were selected. The experimental steps were as follows:

First, cells were seeded in a 96-well culture plate (1 × 10^4^ cells per well) and incubated in a cell culture incubator at 37 °C for 24 h. Second, after 24 h of incubation, we added 25, 50, 100, and 200 μg/mL of material culture solution into the wells, and they were then incubated for another 72 h. Third, 50 μl of MTT was added to each well and was incubated for 4 h. After the reaction, 100 μL of DMSO was added to each well, and the color was changed by shaking for 15 min. Finally, the absorbance values were measured on an enzyme immunoassay analyzer (ELISA reader) at 540 nm. The ability of cells to reduce MTT can be used as an indicator of cell viability. The cell survival rate is calculated as follows:

$${\text{Cell viability }} = {\text{ OD54}}0 \, \left( {\text{test group}} \right)/{\text{OD54}}0 \, \left( {\text{control group}} \right) \, \times { 1}00\%$$

#### Selectivity index (SI)

In the present study, SI = lC50 of pure compound in a normal cell line/LC50 of the same pure compound in cancer cell line, where lC50 is the concentration required to kill 50% of the cell population.

### Statistical analysis

Data were analyzed by using Microsoft Excel 2019. All experiments were performed in triplicate and the results were the average value of three independent experiments. Measurements were presented as means ± standard deviation. A probability value of *P* < 0.05 was considered as statistically significant.

## Results and discussion

### Properties activity analysis of fucoidan

The *S. ilicifolum* samples from different locations came from Pingtung-Kenting, Pingtung-Tanziwan, Pingtung-Nanwan, Pingtung-Chuanfan Rock, Pingtung-Haikou and Taitung-Fugang. The algae from the above five locations were collected in March 2018, and fucoidan was then obtained by hot water extraction (at 65 and 80 °C). From Table1, the results of the yield, it can be seen that the yield gradually increases as the extraction increase, this is because higher extraction temperature breaks cell wall and dissolves cytoplasm, also increases the solubility of polysaccharide, which is consistent with the study of Zhao^35^; Moreover, we also found that the molecular weight of fucoidan extracted from five locations is below 5 kDa. In 2009, Yoon reported that fucoidan is usually divided into low (< 10 kDa), medium (10–10,000 kDa) and high (> 10,000 kDa) molecular weights^21^, according to this, fucoidan in this study belongs to the low molecular weight. The lower the molecular weight and the higher the degree of sulfation, the sulfate content was positively correlated with the antitumor ability^36^, in 2018, Yang reported that low molecular weight fucoidan exhibits anticancer activity^37^. Some sugars may be degraded during extraction, but during the experiment we obtained fucoidan by precipitation, so those sugars should not be precipitated. In the subsequent molecular weight experiments, the molecular weight of crude fucoidan still falls within the range of the calibration curve. In Huang's study in 2015, crude fucoidan was also used for molecular weight analysis. The analysis method was the same as this study, using size exclusion HPLC column and refractive index detector to analyze its molecular weight under different flow rates and mobile phases^38^. From the results of sulfate determination, it can be found that with the increase of extraction temperature, the content of sulfate tends to decrease, it is presumed that some sulfates are damaged due to the increase of extraction temperature. These results are consistent with the study of Ale et al.^39^. From the results of the total sugar determination, the total sugar amounts gradually increased as the extraction temperatures increased. It is because higher temperature led to more sugar release, also total sugar content increased. Based on the results in Table 1, we also found that the total sugar measured for the Taitung-Fugang (A05) location was relatively low compared to the other four regions. It is speculated that Taitung-Fugang belongs to a harbor area, the water is relative deep, while the shallow water area belongs to the reef, means that the brown algae on the rocky coast can directly be illuminated by sunlight at low tide so that it can generate more fucose.

**Table 1:** Yield, sulfate, total sugar, and molecular weight results of fucoidan.

Collection location, number	Temperature (°C)*	Yield (%)*	Molecular weight (kDa)*	Sulfate (wt %)*	Total sugar (wt %)*
Tanziwan, Pin-tong (A01)	65	1.47 ± 0.11	4.79 ± 0.04	28.7 ± 1.00	64.4 ± 5.80
	80	2.47 ± 0.41	4.58 ± 0.10	28.1 ± 0.60	66.1 ± 2.50
Nanwan, Pin-tong (A02)	65	2.53 ± 0.30	4.86 ± 0.10	52.1 ± 0.60	61.7 ± 2.40
	80	2.73 ± 0.23	4.39 ± 0.04	42.7 ± 0.30	66.5 ± 6.20
Chuanfan Rock, Pin-tong (A03)	65	2.33 ± 0.11	4.85 ± 0.11	53.0 ± 1.32	56.6 ± 3.60
	80	2.40 ± 0.20	4.64 ± 0.04	44.3 ± 1.65	70.3 ± 4.50
Haikou, Pin-tong (A04)	65	1.46 ± 0.11	4.97 ± 0.04	42.8 ± 0.41	52.7 ± 3.40
	80	2.20 ± 0.20	4.66 ± 0.10	42.6 ± 1.79	63.9 ± 6.80
Fugang, Taitung (A05)	65	1.40 ± 0.04	4.98 ± 0.04	41.1 ± 1.93	20.3 ± 0.11
	80	1.60 ± 0.07	4.59 ± 0.04	42.6 ± 0.86	20.9 ± 0.22

*Each value represents the mean ± SD of three determinants of the obtained fractions.

### Monosaccharide composition determination using HPLC

To determine whether the fucoidan samples that were extracted at 65 or 80 °C had different monosaccharide compositions, HPLC analysis can determine their monosaccharide compositions. Fucoidan that was extracted by hot water at 65 °C exhibits more biologically active, thus it was selected for further analysis, the results are shown in Table 2. After calculation, the monosaccharide compositions of *S. ilicifolum* from different locations was obtained. Fucose is the major sugar composition in fucoidan, followed by galactose, these results are consistent with Zhang et al. from 2009^1^. From the results in Table 2, the fucoidan from four locations contains more fucose^1,40^, except for the *S. ilicifolum* (A03), and it is inferred that the algae in this location are relatively young, so their fucose contents are relatively low. In addition, we also found that the monosaccharide compositions of fucoidan from different locations are different because fucoidan is a mixture of polysaccharides of different sizes, and its structure and monosaccharide composition is affected by algae species, collection season, age of seaweed and location^41^.

**Table 2:** Monosaccharide composition identification results of fucoidan.

Monosaccharide	Mannose (ppm)*	Glucose (ppm)*	Galactose (ppm)*	Xylose (ppm)*	Fucose (ppm)*
Tanziwan, Pin-tong (A01)	46.36 ± 2.07	7.58 ± 0.07	93.23 ± 2.00	18.42 ± 0.34	112.51 ± 4.88
Nanwan, Pin-tong (A02)	11.57 ± 1.31	19.48 ± 3.22	58.10 ± 1.53	11.20 ± 0.88	104.44 ± 3.09
Chuanfan Rock, Pin-tong (A03)	83.43 ± 0.34	147.41 ± 0.40	116.44 ± 0.16	86.69 ± 0.06	51.49 ± 0.97
Haikou, Pin-tong (A04)	27.63 ± 1.35	19.08 ± 1.57	76.20 ± 3.34	16.53 ± 0.69	137.59 ± 2.17
Fugang, Taitung (A05)	46.97 ± 1.48	21.61 ± 0.69	100.66 ± 2.86	20.37 ± 1.36	100.66 ± 2.86

*Each value represents the mean ± SD of three determinants of the obtained fractions.

### Fourier transform infrared spectroscopy (FT-IR) analysis of fucoidan

**Figure 1:**
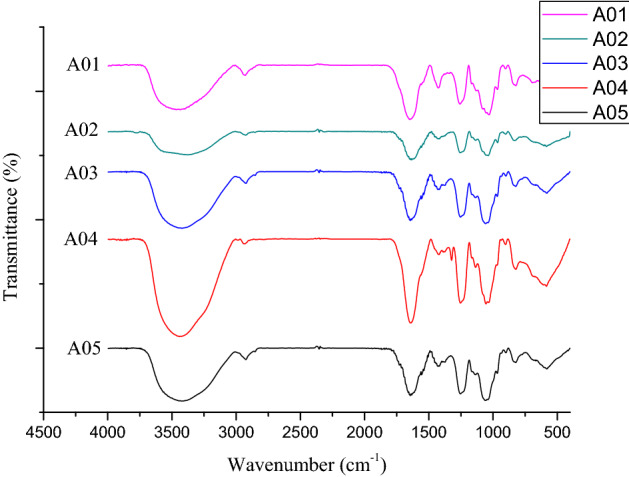
FT-IR spectra of fucoidan from different *S. illicifolium* locations.

It has not been confirmed whether there are differences in the functional groups of fucoidan extracted with hot water in different location. Therefore, in this study, the FT-IR of fucoidan extracted with hot water at 65 and 80 °C was analyzed respectively, the biological activity value extracted at 65 °C was better, thus its FT-IR analysis results were present in Fig. 1. The peak at 3425 cm^−1^ and 2960 cm^−1^ represents the O–H and C–H group of the polysaccharide, the C=O and S=O peak of sulfated polysaccharides at 1641 cm^−1^, 1420 cm^−1^, 1,260 and 1056 cm^−1^, respectively. The peak at 802 cm^−1^ represents C−O−S, which confirms the existence of a sulfate group and thus, the results of this study are consistent with the literature^42^.

### Analysis of polyphenols

The polyphenol yield of fucoidan is shown in Table 3. According to the yield of polyphenols, as the extraction temperature increases, the content of polyphenols increases, this is because solubility increases with temperature, thus more polyphenols can be obtained. In 2022, AT et al. showed that the content of total phenols was increased at high temperature^43^. In addition, the polyphenols of the algae from different locations were also different after extractions at 65 and 80 °C. It was also found that the polyphenol yields of *S. ilicifolium* (A05) were 91.70 ± 8.26 µg g^−1^ and 127.59 ± 4.68 µg g^−1^ at 65 °C and 80 °C, respectively.

**Table 3:** The polyphenol yield of fucoidan.

Collection location, number	Temperature (°C)	Polyphenol (µg g^−1^)*
Tanziwan, Pin-tong (A01)	65	30.59 ± 0.11
	80	94.95 ± 0.41
Nanwan, Pin-tong (A02)	65	47.84 ± 0.30
	80	119.09 ± 0.23
Chuanfan Rock, Pin-tong (A03)	65	93.53 ± 0.11
	80	119.82 ± 0.20
Haikou, Pin-tong (A04)	65	80.62 ± 0.11
	80	101.65 ± 0.20
Fugang, Taitung (A05)	65	91.70 ± 0.04
	80	127.59 ± 0.07

*Each value represents the mean ± SD of three determinants of the obtained fractions.

### Effect of fucoidans on antioxidant activities

**Figure 2:**
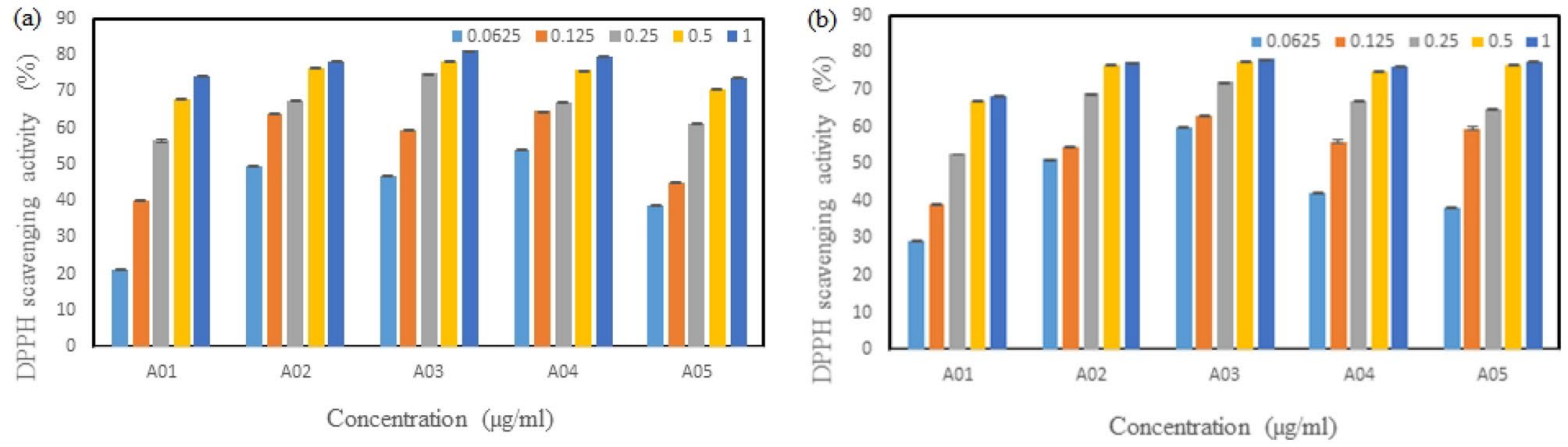
DPPH radical scavenging activity of fucoidan obtained from *S. ilicifolium* at different locations that was extracted at (**a**) 65 °C and (**b**) 80 °C. Bars indicate SD, n = 3.

The scavenging activity of DPPH free radicals of fucoidan is shown in Fig. 2, and we can see that the fucoidan scavenging activity gradually increased as the concentration increased, as shown in Fig. 2. Additionally, the antioxidant activity of the algae from different locations was also different after extractions at 65 and 80 °C. It was also found that the antioxidant activities of *S. ilicifolum* (A03) at 65 and 80 °C were 81 and 78% at concentrations of 1 mg/mL, respectively. From the polyphenol experiments, it can be known that the polyphenol content of *S. ilicifolium* (A03) at 65 °C. is better than the other four location, and the polyphenol content is also related to the antioxidant activity. It was confirmed that *S. ilicifolum* in these five locations exhibits antioxidant activity.

**Figure 3:**
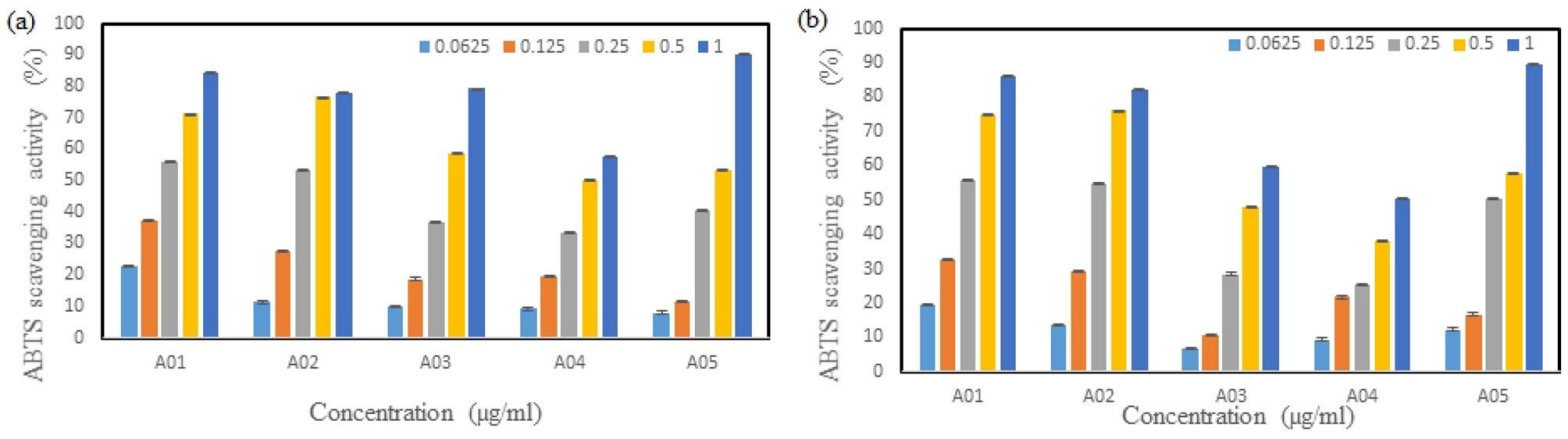
ABTS radical scavenging activity of fucoidan obtained from *S. ilicifolium* at different locations that was extracted at (**a**) 65 °C and (**b**) 80 °C. Bars indicate SD, n = 3.

The scavenging activity of ABTS free radicals of fucoidan is shown in Fig. 3, and we can see that the scavenging activity of fucoidan gradually increased as the concentration increased, as shown in Fig. 3. Additionally, the antioxidant activities of the algae from different locations were also different after extractions at 65 and 80 °C. It was also found that the antioxidant activities of *S. ilicifolum* (A05) at 65 and 80 °C were 89 and 90% at concentrations of 1 mg/mL, respectively. The results of polyphenol experiments exhibits that the polyphenol content of *S. ilicifolium* (A05) is superior than other four regions, and the polyphenol content is also related to the antioxidant activity. These results confirmed that *S. ilicifolum* from these five locations exhibits antioxidant activity again.

**Figure 4:**
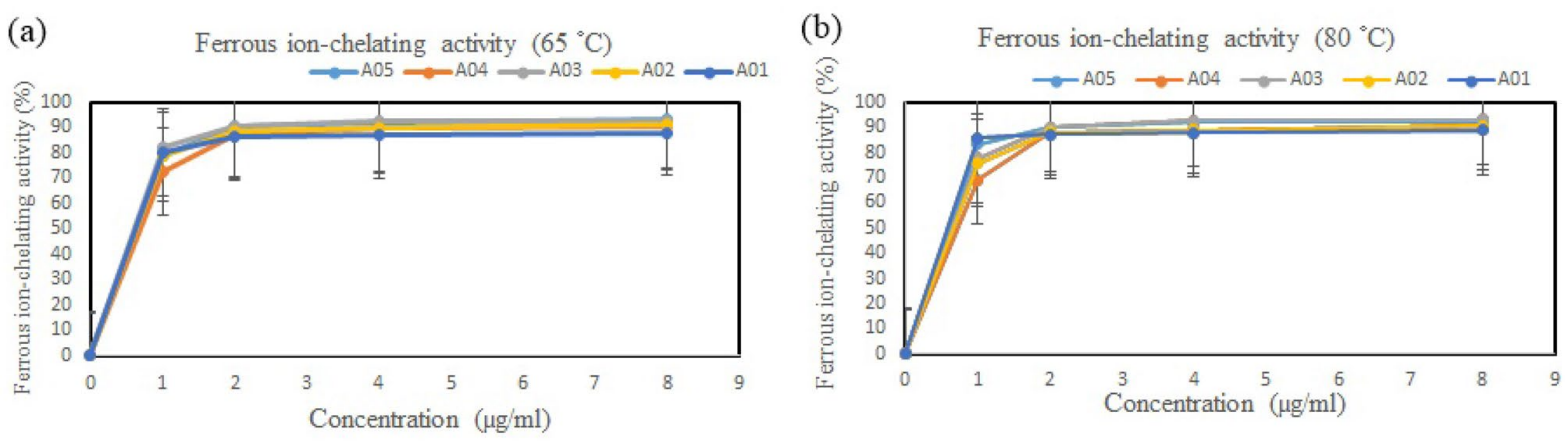
Ferrous ion-chelating activity of fucoidan obtained from *S. ilicifolium* at different locations that was extracted at (**a**) 65 °C and (**b**) 80 °C. Bars indicate SD, n = 3.

The ferrous ion-chelating activity of fucoidan is shown in Fig. 4, and we can see that the scavenging activity of fucoidan gradually increased as the concentration increased, as shown in Fig. 4. Additionally, the antioxidant activities of the algae from different locations were also different after extraction at 65 and 80 °C. It was also found that the antioxidant activities of *S. ilicifolum* (A05) at 65 and 80 °C were 93 and 92% at concentrations of 1 mg/mL, respectively. The results are also related with the polyphenol experiments.

**Figure 5:**
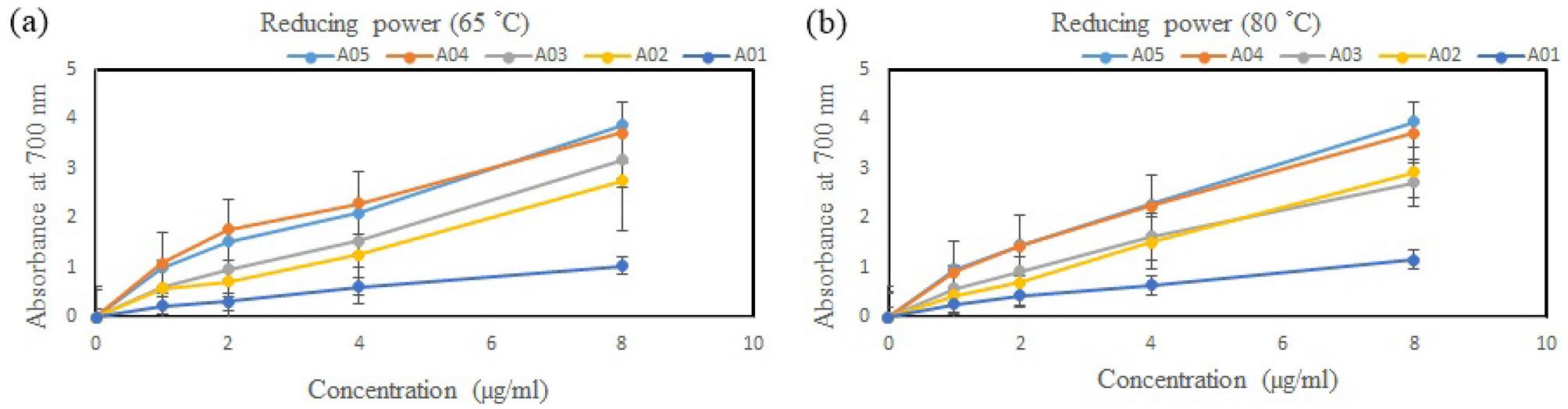
Reducing power of fucoidan obtained from *S. ilicifolium* at different locations that was extracted at (**a**) 65 °C and (**b**) 80 °C. Bars indicate SD, n = 3.

Figure 5 depicts the reducing ability of fucoidan for the five locations. All extracts exhibit a dose-dependent reduction ability. A05 has the strongest reducing power, which is followed by A04 and A03 and then A02 and A01. Studies have shown that for the reducing power of fucoidan that was extracted from *S. ilicifolum* (A05) at 65 and 80 °C, when the concentrations were 2 mg/ml, the educing powers were approximately 1.526 and 1.45. Figure 5 indicate that the sulfate/fucose ratios of the polysaccharides may affect antioxidant activity, this result is similar with the three previous antioxidation tests. Generally, the crude extract of fucoidan (A05) from *S. ilicifolum* showed the most excellent antioxidant activity among the four current antioxidant studies.

### Effect of fucoidans on anticancer activities

**Figure 6:**
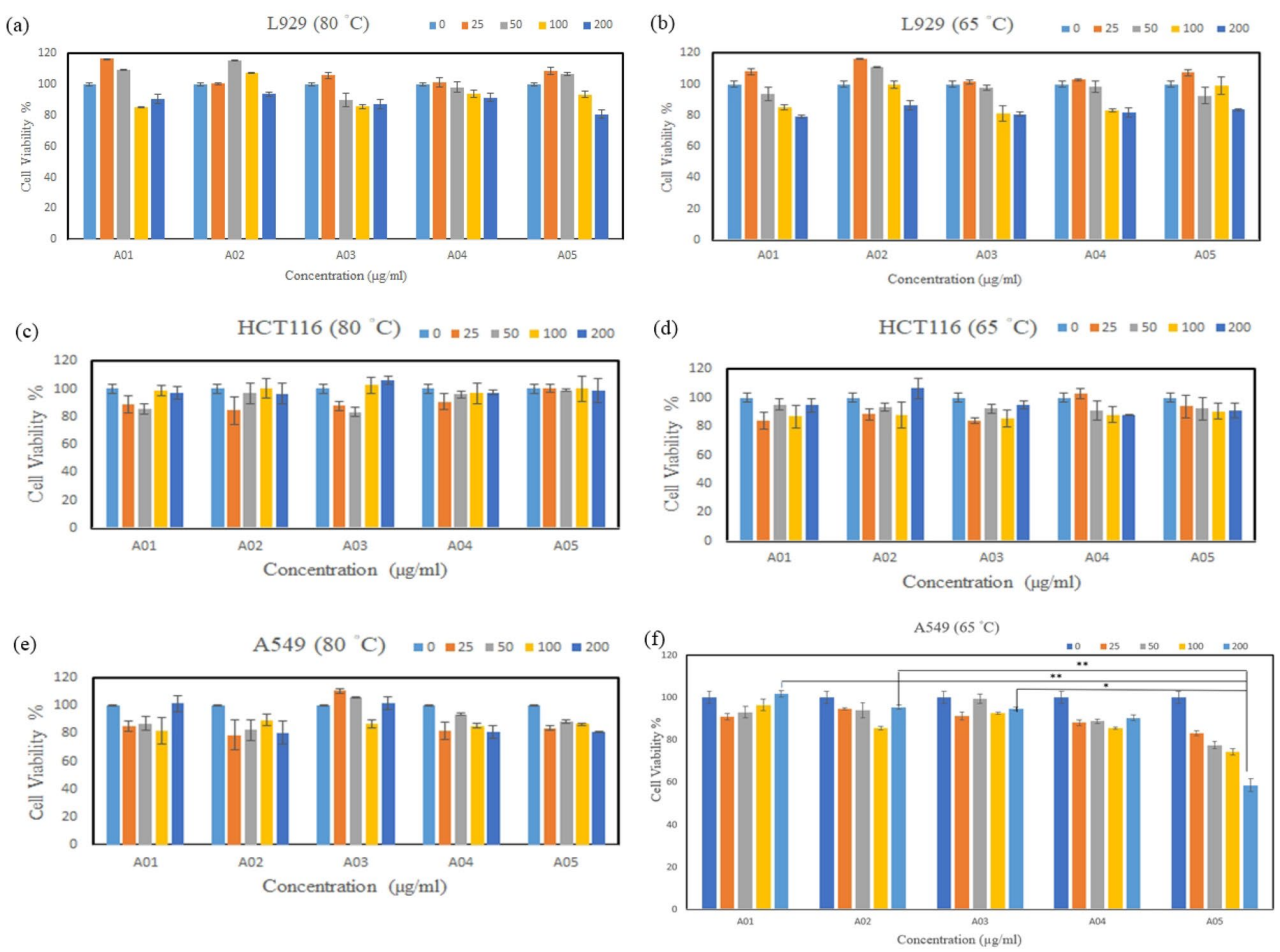
Fucoidan of *S. ilicifolium* from different locations that was extracted at (**a**) 65 °C and (**b**) 80 °C on the cell viability of the L929 (**a**) (**b**), HCT116 (**c**)(**d**) and A549 (**e**)(**f**) cell lines. Bars indicate SD, n = 3. (**P < 0.01 and *P < 0.05).

To examine the effects of fucoidan on normal and cancer cells, cell viability tests were performed. The results in Fig. 6a, b, c, d, e, f show that fucoidan that was extracted at both temperatures did not affect the cell viability of the normal cell line L929. However, with regard to the cancer cell lines A549 and HCT116, cancer cell viability remained over 80% at all concentrations (e.g., 0, 25, 50, 100, and 200 μg/ml), except for Taitung-Fugang (A05). Fortunately, fucoidan extracted from the Taitung-Fugang (A05) location at 65 °C exhibited obvious cytotoxicity to A549 cells at a concentration of 200 μg/mL, and the cell viability remained at 58% after treatment, while the same fucoidan extracted at 80 °C had little effect on A549 cells. It is speculated that high-temperature extraction leads to sulfate linkages, and the reduction of sulfate affects the cytotoxicity of fucoidan. Since selectivity index (SI) indicate different activities of pure compounds, the larger the SI value, the stronger the selectivity. Compounds with SI values < 2 are considered moderately toxic and may also cause cytotoxicity in normal cells. In 2009, Bardisa literature studied the selective cytotoxic activity of two new drugs on human breast cancer MCF-7 cells, in which the si of piperidine-DES and pyrrolidinyl-DES were 1.78 and > 2.84, respectively. The pyrrolidinyl-DES possesses has strong selectivity and strong anticancer activity. Interestingly, 4-hydroxytamoxifen, widely used as an antiestrogen therapy in human breast cancer, has an SI of < 2. Compared with this study, although the SI of the extracted fucoidan was 0.96, which was inferior to that of the pyrrolidinyl-DES synthesized by Bardisa, the SI were similar to those of the other two groups of data (piperidine-DES and 4-hydroxyl), which were all less than 2. Therefore, it can be seen from the results that although the selectivity and anticancer properties of natural product drugs are not as good as those of chemically synthesized drugs, they still have good effects^44^.

## Conclusion

In this study, we extracted fucoidan from *S. ilicifolium* samples that were collected from five different locations in southern Taiwan and were extracted at two temperatures, 65 and 80 °C; after further examinations by DPPH, ABTS, ferrous ion chelating, reducing power and cell viability tests, only the 65 °C extraction from the Fugang-Taitung location exhibited anticancer ability and good antioxidation ability. Therefore, it is expected that this drug can be used in drug delivery systems.

## Data Availability

The datasets used during the current study available from the corresponding author on reasonable request.
